# Rudimentary Organs

**Published:** 1889-12

**Authors:** 


### RUDIMENTARY ORGANS.

One of the strongest confirmations of the Darwinian theory of descent is found in the existence of rudimentary organs in animals, which, while subserving no useful purpose, still exist as an inheritance from some lower form of life to which they were both useful and necessary.

One of the most familiar examples in the human body is that of the nails. These appendages are really of no particular use, and render no service in the struggle for existence. Artificial uses have been found for them, it is true ; we could hardly open our pen-knives without their aid, but this is a case where their previous existence has suggested a subsequent use. The nails suggested the common type of knife-blade, but they were certainly not developed through many generations of knife-opening beings, with the survival of those best fitted to open their knives with promptness in case of trouble. It is only when we look back among the lower animals, and see how important a part of their means of offense and defense the nails become, that we realize that our digital appendages are simply the useless degenerate forms of those which, in some very remote progenitor, were of the greatest importance to his comfort and safety.

The hair may be considered as another worthless inheritance of ours, and the occasional cases where it grows quite thickly upon the body as a partial reversion to some former type. It is a curious fact that it still grows luxuriantly upon the head, where it is of very little use, and its presence there is hard to be explained, except by considering it as an ornamental appendage, like the brilliant plumage of birds, thus bringing into play the principle of sexual selection. The fact that women rarely become bald adds weight to this supposition.

One of the most curious instances of a rudimentary organ is to be found in the *cæcum*, a portion of the intestines, which is in shape like a small pouch, attached to the alimentary canal. It subserves no use whatever in the human organism, and is, in fact, a source of danger, for, although the opening is closed by a wonderfully constructed valve, yet fatal accidents have occurred from foreign matters passing into it, and inducing inflammation and abscesses. If we examine the intestines of a herbivorous animal, such as an ox, we find the same cæcum present, but greatly enlarged, and usually filled with partly digested food. In these animals it is undoubtedly an important part of the digestive apparatus, but only survives in man as a useless and dangerous appendage.

Certain valves in the veins of man are very poorly adapted to support the blood pressure due to an upright position, and were probably first developed in some animal which walked upon all four limbs, and it has recently been discovered that the portal veins of newly born infants contain rudimentary valves, which soon disappear, but which are always present in some of the lower animals during their entire life.

Rudimentary organs are found in other animals as well as man. The clavicle, or collar-bone, of the cat is a small bone, apparently of little consequence, but in man it is a most important means of maintaining an outward position of the shoulder-joint, so as to allow the widest range of motion to the limb. The "jew-claws" which hang down from the feet of deer, and the splint bones in a horse's leg, are well-marked survivals of a previously useful member.

The occasional occurrence of uncommon organs in the human body may rather be considered as cases of reversion to former types, than as direct survivals. The power possessed by certain persons of moving the ears and scalp, is an instance of this, as well as the hairy men and women, and many other dime museum "freaks."

There are certain organs of which the use is still unknown, such as the supra-renal capsules or the prostate gland, which may or may not be survivals. It is more probable that they serve some unknown purpose in the human economy, especially as when attacked by disease they often produce severe constitutional disturbance.

The presence of rudimentary organs is, however, a settled fact, and is a strong argument against the distinct and separate origin of the different forms of life. It is impossible to think that the superfluous and dangerous cæcum would have been introduced into the human anatomy if man had come into existence independently of those forms in which it is an important organ, and it is a belittling conception of a Creator which regards him as forming his special creations in weak imitation of each other, and endowing them with useless organs and members, without regard to the varying conditions of their future environment.—*Popular Science News*.

